# Rapid Screening and Analysis of Putative Bioactive Candidates in Zhizhu Decoction Using Membrane-Based Affinity Ultrafiltration-Mass Spectrometry

**DOI:** 10.3390/membranes16100322

**Published:** 2026-09-27

**Authors:** Shujun Wang, Renai Fu, Xiaowen Hua, Yongbing Xu, Yingying Zhang, Mingquan Guo

**Affiliations:** 1 Affiliated Cixi Hospital, Wenzhou Medical University, Cixi 315300, China; m13429332988@163.com; 2 Zhejiang International Scientific and Technological Cooperative Base of Biomedical Materials and Technology, Zhejiang Engineering Research Center for Biomedical Materials, Ningbo Cixi Institute of Biomedical Engineering, Cixi 315300, China; furenai@nimte.ac.cn (R.F.); huaxiaowen@nimte.ac.cn (X.H.); xuyongbing@nimte.ac.cn (Y.X.); 3 Laboratory of Advanced Theranostic Materials and Technology, Ningbo Institute of Materials Technology and Engineering, Chinese Academy of Sciences, Ningbo 315201, China

**Keywords:** Zhizhu Decoction, hypolipidemic candidates, hesperidin, naringin, affinity ultrafiltration

## Abstract

The Zhizhu Decoction (ZZD), as a classic traditional Chinese medicine prescription, has long served as an effective treatment for obesity. However, rapid analysis of its putative bioactive components and their mechanisms of action remains a challenging task due to its chemical complexity and a lack of target-based screening strategies. This study established a rapid screening system using ultrafiltration membrane-based bio-affinity ultrafiltration-mass spectrometry (AUF-MS), combining lipase and α-glucosidase for targeted screening. Ultrafiltration centrifuge tubes made of regenerated cellulose membrane material were used as the core separation unit: following incubation of the enzymes with ZZD, the ultrafiltration membrane preferentially retained compounds in the presence of the target enzymes, while unbound free small molecules were removed through multi-stage buffer centrifugation and washing. Subsequently, compounds showing preferential retention in the presence of the target enzymes were eluted and further characterized using ultra-performance liquid chromatography quadrupole time-of-flight tandem mass spectrometry (UPLC-Q-TOF-MS/MS). Candidate bioactive compounds were further evaluated in a free fatty acid (FFA)-induced lipid deposition cell model. We detected 39 compounds in the ZZD extract, and through an ultrafiltration membrane screening system, at least 12 compounds showing preferential retention in the presence of lipase were successfully putatively identified. Notably, naringin, hesperidin, and poncirin showed preferential retention in the lipase-based AUF-MS screening, and their potential cellular lipid-regulating effects were further evaluated in a free fatty acid (FFA)-induced lipid deposition cell model, where all three compounds were associated with reduced lipid accumulation and triglyceride content under the tested conditions. In particular, naringin and hesperidin showed notable effects at relatively low concentrations, highlighting their potential as promising bioactive compounds for further investigation. The study identified several putative enzyme-interacting candidates from ZZD using a membrane-based AUF-MS screening strategy, and selected compounds were further evaluated in an FFA-induced HepG2 cell model for their potential lipid-regulating effects. More importantly, it demonstrated the feasibility of applying the screening strategy for preliminary candidate identification.

## 1. Introduction

Due to shifts in lifestyles around the world, the rate of lipid metabolism disorders and related diseases has surged, placing a significant burden on public health. Citing IDF statistics, by 2050, the global prevalence of diabetes will reach 12.96%; in absolute terms, the health of approximately 853 million people will be affected [1]. Metabolic Dysfunction-Associated Steatotic Liver Disease (MASLD) exhibits a worldwide prevalence of 30–40%, representing 60–70% of type 2 diabetes cases and 70–80% of obesity cases [2]. Obesity and related metabolic disorders have become major global health challenges, driven by chronic energy imbalances and persistent low-grade inflammation involving immune cell polarization and metabolic reprogramming [3]. Although some Western medications can effectively control blood glucose and lipid levels, they are often associated with side effects. Common side effects include liver damage, acute kidney injury, and insulin resistance [4,5]. And their long-term efficacy may be limited. Therefore, there is an urgent need for new sources of drugs to effectively intervene in and treat these chronic diseases involving disorders of glucose and lipid metabolism. Accumulating evidence highlights that natural product constituents and functional food-derived metabolites play pivotal roles in regulating metabolic functions, offering a rich repository for bioactive lead discovery [6].

Traditional Chinese medicine (TCM) has a long history and some unique advantages when it comes to preventing and treating chronic metabolic diseases. Zhizhu Decoction (ZZD), which originates from the classical Chinese medical text “Jin Gui Yao Lue”, is made up of two medicinal materials: *Citrus aurantium* L. and *Atractylodes macrocephala* Koidz. In traditional medicine, ZZD is primarily used to treat abdominal distension, abdominal fullness, and indigestion; however, its modern applications have expanded to include the management of symptoms associated with metabolic disorders, including diseases related to glucose and lipid metabolism. Data from pharmacological assays confirmed that Zhizhu granules reduced the levels of total cholesterol (T-CHO), triglycerides (TG), low-density lipoprotein cholesterol (LDL-C), and glucose (GLU) in diseased rats, The treatment also improved hepatic steatosis and partially restored the metabolic imbalances [7]. Individual medicinal herbs in ZZD have also demonstrated the ability to regulate lipid metabolism, alleviate liver injury, and improve obesity. *C. aurantium* exerts significant hepatoprotective effects against acetaminophen (APAP)-induced liver necrosis. This effect may be related to the inhibition of p53 up-regulated modulator of apoptosis (PUMA) and the restoration of hepatic lipid homeostasis [8]. *A. macrocephala* may help alleviate hyperlipidemia by improving the function of serum high-density lipoprotein cholesterol (HDL-C). It may promote the reverse cholesterol transport (RCT) process through the transcriptional activation of related proteins. These proteins include apolipoprotein A-I, lecithin-cholesterol acyltransferase, and scavenger receptor class B type I [9]. Although ZZD has been reported to have hypolipidemic effects, its specific pharmacodynamic material basis remains insufficiently clarified.

TCM often poses challenges in identifying its active ingredients. Efficiently screening active ingredients from complex TCM systems is a key step in TCM modernization. Most of the conventional phytochemical screening techniques developed in the past often rely on sequential extraction, fractionation, purification, and activity testing, which can be laborious and time-consuming, and tend to overlook weakly active compounds. Affinity ultrafiltration technology has gradually gained attention. It has now become an effective method for isolating and purifying bioactive constituents in TCM [10]. Regarding current bioaffinity screening technologies, several methods are gradually gaining attention, including surface plasmon resonance (SPR), affinity chromatography (AC), and magnetic bead-based target separation techniques [11,12,13]. SPR and AC provide useful approaches for studying molecular interactions. However, both methods require complex target immobilization. This process may cause changes in protein conformation and steric hindrance, and it may also be affected by matrix interference. The bioaffinity ultrafiltration (AUF) method is primarily based on the principle of target–ligand interactions and uses semipermeable ultrafiltration membranes to screen for potential ligands. The ultrafiltration membrane functions as a molecular sieve. It preferentially retains compounds in the presence of the target enzyme, whereas unbound small molecules can pass through freely. This study developed a multi-target affinity ultrafiltration-mass spectrometry (AUF-MS) method that uses disease-related target proteins for screening and can rapidly and effectively identify potential bioactive compounds [14,15,16]. The advantage of this method is that it directly exploits target–ligand interactions to enrich potential bioactive compounds from complex ZZD extracts, followed by mass spectrometry-based identification. This reduces the reliance on extensive preliminary separations and enables rapid screening of potential bioactive compounds based on their preferential retention in the presence of preselected targets.

This study innovatively combines multi-target screening, flexible membrane selection, and a cell model to establish a rapid and comprehensive platform for the preliminary screening of potential bioactive components in TCM. The membrane can be selected according to the molecular size of the target enzyme, allowing the method to be readily adapted to different targets. In the present study, the ZZD extract was subjected to multi-target affinity ultrafiltration using α-glucosidase and lipase as the target enzymes, and then the retained potential active compounds were identified by UHPLC-Q-TOF-MS/MS. Subsequently, the screened components were further validated for their potential effects on glucose and lipid regulation using free fatty acids (FFA)-induced lipid-loaded cell model. This provides further information on the potential bioactive components of ZZD and provides a basis for further investigating its effects on glucose and lipid metabolism. Furthermore, it demonstrates the effectiveness and application value of the multi-target AUF-MS method in the discovery of active ingredients from TCM.

## 2. Materials and Methods

### 2.1. Plant Material

ZZD was prepared from a traditional Chinese medicine formula composed of *C. aurantium* (12.0 g) and *A. macrocephala* (6.0 g). The dried *C. aurantium* (Batch No. 240504561, originating from Hunan Province, China) and *A. macrocephala* (Batch No. 240500269, originating from Anhui Province, China) were purchased from Kangmei Pharmaceutical Co., Ltd. (Shenzhen, China) on 14 August 2024. All medicinal botanical batches complied with the prescribed quality criteria outlined in the Chinese Pharmacopoeia (2020 revision) and were authenticated by Professor Guo Mingquan of the Ningbo Institute of Materials Technology and Engineering, Chinese Academy of Sciences. Voucher specimens (*A. macrocephala*, 2024BZ1–2024BZ3; *C. aurantium*, 2024ZS1–2024ZS3) were deposited at the Marine Biomedical Materials and Technology Laboratory, Ningbo Institute of Materials Technology and Engineering, Chinese Academy of Sciences.

### 2.2. Preparation of Sample Extracts

ZZD was prepared from precisely weighed amounts of *C. aurantium* (60.0 g) and *A. macrocephala* (30.0 g). All the herbs were soaked in 10 times their weight in purified water for 30 min. Then, herbs were boiled for 1 h. The liquid from the decoction was allowed to cool to room temperature, then centrifuged to collect the supernatant. Decocting the remaining herbal residue was continued in 8 times its weight of purified water. The supernatant was collected again. Finally, the two supernatants were combined, and the ZZD extract was obtained through processes such as rotary evaporation and freeze-drying.

### 2.3. Analysis of Cell Viability

To assess cell viability using the Cell Counting Kit-8 (CCK-8) assay (UElandy Inc., Suzhou, China), hepatoma G2 (HepG2) cells in the logarithmic growth phase were digested with trypsin and subsequently collected. The HepG2 cell line used in this study was kindly provided by Dr. Fuqiang Song from Wenzhou Medical University. It was originally obtained from the Cell Bank of Typical Culture Collection of the Chinese Academy of Sciences (Shanghai, China; Catalog No. SCSP-510). The cells were received at passage 12 and were used for experiments between passages 12 and 15. HepG2 cells were cultured in high-glucose Dulbecco’s Modified Eagle’s Medium (DMEM) (Sangon Biotech, Shanghai, China) supplemented with 10% fetal bovine serum (FBS) (Sangon Biotech, Shanghai, China) and 1% penicillin-streptomycin (Solarbio, Beijing, China). Cells were plated in 96-well plates at a concentration of 5000 cells per 100 μL and cultured at 37 °C in a humidified atmosphere containing 5% CO_2_ for at least 12 h. After the cells attached well, the culture medium was replaced with 100 μL of fresh medium supplemented with various concentrations of the test compounds. Naringin, hesperidin, and poncirin were dissolved in pure DMSO (Macklin, Shanghai, China) as stock solutions and subsequently diluted with complete medium to achieve a final working DMSO concentration of 0.1%. To eliminate any potential solvent-induced interference, the control group containing the same final concentration of DMSO (0.1%) without the test compounds was included. Subsequent cell-based assays for these three compounds followed a similar dissolution protocol. Blank wells containing only medium without cells were prepared at the same time. After 24 h of treatment, 10 μL of CCK-8 reagent was added to each well, and the plates were incubated at 37 °C for another 1 h after being sealed to reduce evaporation, then the absorbance at 450 nm was recorded.

### 2.4. Analysis of Lipid Metabolism Parameters

Exponentially growing HepG2 cells were distributed into 6-well plates at 1 × 10^6^ cells per well. After the cells attached overnight, the cells were washed twice with PBS (Sangon Biotech, Shanghai, China) and then treated with different treatments. The cells in the control group received normal complete culture medium. For the ZZD treatment experiment, the positive control was incubated with Shenqi Jiangtang Granules (SQJT, Lunan Pharmaceutical Group, Linyi, China). The main constituents of SQJT are *Ginseng folium* et caulis total saponins, *Schisandrae fructus*, *Astragali radix*, *Dioscoreae rhizoma*, *Rehmanniae radix*, *Rubi fructus*, *Ophiopogonis radix*, *Poria*, *Trichosanthis radix*, *Alismatis rhizoma*, and *Lycii fructus*. SQJT was used at a concentration of 250 µg/mL in the assay. The remaining groups received different concentrations of the ZZD and were cultured for another 24 h. After the treatment, the culture supernatant was collected. Glu, TG, and T-CHO were measured in the culture medium collected after cell treatment using commercial assay kits (Nanjing Jiancheng Institute of Bioengineering, Nanjing, China) and expressed as mmol/L according to the assay procedures for liquid samples. For the subsequent cellular validation experiments with naringin, hesperidin, and poncirin, the positive control was incubated with orlistat. Orlistat was purchased from Shanghai Yuanye Bio-Technology Co., Ltd. (Shanghai, China) (Cat. No. S69030; Batch No. JS236780). Orlistat and the tested compounds were dissolved in pure DMSO as stock solutions and subsequently diluted with complete culture medium to achieve the required working concentrations, with a final DMSO concentration of 0.1%. The control group containing the same final concentration of DMSO (0.1%) without the test compounds was included. Orlistat was used at a final concentration of 250, 25 and 10 μg/mL as the positive control for naringin, hesperidin, and poncirin, respectively. Reference standards of naringin (Cat. No. N814677, Lot No. C138291248, purity 95%) and hesperidin (Cat. No. H810891, Lot No. KS415987, purity 95%) were purchased from Shanghai Macklin Biochemical Co., Ltd. (Shanghai, China). Poncirin (Cat. No. NP02807, Lot No. FR250301, purity ≥ 98%) was obtained from Shanghai Fuorain BioTech Co., Ltd. (Shanghai, China). Glu and TG were measured in HepG2 cell lysates. Total protein concentration was determined using the BCA Protein Concentration Assay Kit (Sangon Biotech, Shanghai, China; Cat. No. C503061-1250). The glucose and TG contents were normalized to the corresponding total protein content and expressed as mmol/g protein. For the Glu (Cat. No. A154-2-1), TG (Cat. No. A110-1-1) and T-CHO (Cat. No. A111-1-1) assays, the absorbance values of the blank, standard, and sample wells were measured according to the manufacturer’s instructions, and the concentrations were calculated accordingly.

### 2.5. Oil Red O Staining

For the lipid accumulation experiment, exponentially growing HepG2 cells were plated in 6-well plates at a density of 1 × 10^6^ cells per well, in 2 mL of medium. The cells were cultured under standard culture conditions (37 °C, 5% CO_2_) for 12 h. After confirming that the cells adhered well, the medium was removed, and 2 mL of 0.5 mM FFA-BSA solution containing different test compounds was added. For the preparation of the FFA-BSA complex, sodium oleate (OA) and sodium palmitate (PA) were purchased from Macklin (Shanghai, China) and dissolved to prepare 20 mM stock solutions. To facilitate dissolution, the PA solution was heated at 70 °C for 10 min. A 25% (*w*/*v*) BSA (Sangon Biotech, Shanghai, China) solution was prepared in PBS and filtered for sterility. OA and PA were separately conjugated with BSA through mixing and sonication for 30 min. The resulting OA and PA solutions were combined at a 2:1 molar ratio (OA:PA) to yield the FFA stock solution, which was sterile-filtered, aliquoted, and stored at −20 °C. Prior to use, the stock solution was briefly sonicated and diluted with complete medium to a final concentration of 0.5 mM. The plates were kept under normal culture conditions for an additional 24 h to induce lipid accumulation. Following incubation, the medium was aspirated, the cells were washed twice with warm PBS, and subjected to fixation with 4% paraformaldehyde (Solarbio, Beijing, China) for 30 min. The fixative was discarded, and the cells were washed twice with distilled water, followed by incubation with 60% isopropanol for 25 s. After that, freshly prepared Oil Red O staining solution (Solarbio, Beijing, China) was added for 15 min. After discarding the staining solution, the cells were quickly washed with 60% isopropanol for 20 s until a clear background was achieved, washed once with PBS and four times with distilled water until no obvious color remained. The cell nuclei were stained with Mayer’s hematoxylin (Sangon Biotech, Shanghai, China) for 2 min, then the cells were rinsed with tap water. The stained cells were examined using an inverted microscope (Nikon TS2R-FL, Nikon, Tokyo, Japan) at 400× magnification. The scale bar was 50 μm. Three staining wells (biological replicates) were set up for each group. Images were analyzed using ImageJ software (version 1.54k). After conversion to the appropriate color space, the same thresholding criteria were applied to all images to identify Oil Red O-positive areas. The percentage of Oil Red O-positive area was calculated as the stained area divided by the total cell/image area.

### 2.6. Identification of Chemical Components in ZZD by UPLC-QTOF-MS/MS

Accurately weighing 1.0 g of ZZD extract and dissolving it in 15 mL of methanol to obtain a stock solution at 66.7 mg/mL. The stock solution was subsequently diluted with methanol to a final concentration of 40 mg/mL for UPLC-Q-TOF-MS/MS analysis.

For the analytical workflow, an Agilent 1290 UPLC system was used, and it was connected to a 6545 Q-TOF MS detector (Agilent Technologies, Santa Clara, CA, USA). Separation was carried out using an EC-C18 column (3.0 × 150 mm, 2.7 μm), and the column temperature was set to 25 °C. The mobile phase consisted of 0.1% formic acid in water (A) and acetonitrile containing 0.1% formic acid (B). The flow rate was 0.3 mL/min, with an injection volume of 5 μL. For positive ion mode, the gradient started at 5.0% B, then B was linearly increased to 95.0% over 0.00–40.00 min and maintained at 95.0% B for 6.0 min (40.00–46.00 min); the composition was returned to the initial condition (5.0% B) at 46.01 min and held until 52.00 min for column re-equilibration. For negative-ion mode it was set as: initial 5.0% B; linearly increased to 95.0% B over 0.00–40.00 min and maintained at 95.0% B for 10.0 min (40.00–50.00 min); returned to initial conditions (5.0% B) at 50.01 min and held until 60.00 min to re-equilibrate the column. The mass spectrometer was operated in positive and negative electrospray ionization (ESI) modes. The ion-source parameters were set as follows: capillary voltage, 3500 V; drying gas temperature, 350 °C; drying gas flow rate, 8 L/min; and nebulizer pressure, 35 psi. The fragmentor voltage was set at 175 V. Data were acquired in Auto MS/MS mode at 3 spectra/s over an *m*/*z* range of 50–1100. Collision energies of 10, 20, and 40 V were used for MS/MS acquisition. Real-time internal reference mass correction (lock mass) was automatically performed during data acquisition using the Agilent Tune Mix/Reference Ion solution, with reference ions at *m*/*z* 121.0508 and 922.0098 in positive ion mode and *m*/*z* 112.9855 and 1033.9881 in negative-ion mode to ensure continuous mass accuracy calibration. A mass error threshold of <10 ppm was accepted for compound identification and molecular formula generation.

Following chromatographic separation, compounds were putatively identified based on accurate precursor masses, characteristic MS/MS fragmentation patterns, comparisons with the Agilent PCDL (ForensicsTox_AM_PCDL HMDB and LCQTOF Applied Markets PCDL), HMDB databases, and the previously published literature. As authentic standards were not used for individual confirmation, the identified compounds were assigned as Level 2 putative identifications according to commonly used metabolomics identification criteria.

### 2.7. Bio-Affinity Ultrafiltration Screening

A ZZD sample (100 µL, 4 mg/mL) was incubated with α-glucosidase (100 µL, 2 U, MW ~67–70 kDa) and lipase (100 µL, 2 mg/mL, MW ~50–52 kDa) for 40 min at 37 °C. α-Glucosidase from yeast (Cat. No. G5003-100UN; Sigma-Aldrich, St. Louis, MO, USA) and lipase from porcine pancreas (Cat. No. S10035-5g; Yuanye Bio-Technology, Shanghai, China) were used in this study. Heat-inactivated enzymes were used as the negative control, and the enzymes were inactivated by heating them in boiling water for 15 min before the incubation step. After incubation, the samples were subjected to ultrafiltration using Amicon^®^ Ultra centrifugal ultrafiltration devices (Sigma-Aldrich, St. Louis, MO, USA). The 30 kDa MWCO device (Cat. No. UFC5030) was used separately for α-glucosidase–ZZD mixtures, whereas the 10 kDa MWCO device (Cat. No. UFC5010) was used separately for lipase-ZZD mixtures. Both devices consisted of a polypropylene cap and tube, a regenerated cellulose membrane, and a styrene-butadiene housing. Centrifugation was performed at 11,000× *g* for 10 min at 4 °C. The retained fraction was washed three times with 200 μL of PBS buffer (pH 6.8) each time, then the samples were centrifuged again under the same conditions to remove unretained compounds. 200 µL of 90% methanol was added to elute the preferentially retained compounds. Following a 10 min standing period, the sample underwent centrifugation at 11,000× *g* for 10 min, after which the eluate was harvested. The elution step was performed twice, and the combined eluates were allowed to dry under a gentle nitrogen stream. An aliquot of 100 µL methanol was added to dissolve the residue before UPLC-Q-TOF-MS/MS analysis. Each experiment was independently performed once.

The bioactive constituents identified by affinity ultrafiltration approach were characterized using an Agilent 1290 UHPLC system and a 6545 Q-TOF mass spectrometer. All instrument parameters were consistent with those provided in Section 2.6. Calculate the binding degree between the active ingredient and the target enzyme according to the following formula: BD (binding degree) = (Peak area of active enzyme − Peak area of inactivated enzyme)/Peak area of active enzyme × 100%.

### 2.8. Statistical Analysis

Measured *m*/*z* values and characteristic ion fragments were analyzed using Qualitative Analysis 12.0 software. Further compound information was recorded, and BD values were calculated using WPS Office. Cellular experiments were performed using at least three independent biological replicates, with no separate technical replicates. Data are presented as the mean ± standard deviation (SD). Normality was assessed using the Shapiro–Wilk test, and homogeneity of variances was assessed using Levene’s test. Statistical significance was assessed by one-way analysis of variance (ANOVA) followed by Dunnett’s multiple-comparison test using GraphPad Prism software (version 10.1.2). A *p* value < 0.05 was considered statistically significant.

## 3. Results

### 3.1. Preliminary Effects of ZhiZhu Decoction on Lipid Metabolism in HepG2 Cells

As indicated by Figure 1, ZZD maintained high cell viability at concentrations up to 1000 μg/mL (Figure 1A), which led to the selection of 500, 250, and 125 μg/mL for subsequent experiments. The positive control, Shengqi Jiangtang Granules, significantly lowered Glu, TG, and T-CHO levels (*p* ≤ 0.05). A similar reduction in Glu, TG, and T-CHO was observed with ZZD at 500 μg/mL. At the concentration of 250 μg/mL, ZZD still produced a significant decrease in Glu (*p* ≤ 0.05), though changes in TG or T-CHO were not apparent. The low-dose group (125 μg/mL) showed no significant effect on these parameters.

### 3.2. Annotation of Chemical Components in Zhizhu Decoction

Analysis using UPLC-Q-TOF-MS/MS in negative-ion mode established the chemical profile of the ZZD extract; 25 compounds were putatively identified, excluding several unidentified constituents (Table 1). These compounds were mainly classified as organic acids, including galactaric acid and vanillic acid, and flavonoids such as hesperidin and poncirin. Table 2 lists the compounds identified in the positive ion mode. Overall, 17 compounds were detected. The identified compounds were predominantly flavonoids, limonoids, and alkaloids. Lonicerin and poncirin serve as examples detected in both positive and negative-ion modes, where their precursor ions showed consistent characteristic MS/MS fragments across the two ionization modes. The results obtained from the two ionization modes complemented each other well, and the combined data increased both the coverage and the reliability of compound identification.

### 3.3. Affinity Ultrafiltration Screening of Potential α-Glucosidase and Lipase-Interacting Components

The potential bioactive components in ZZD with relatively BD values in the presence of α-glucosidase and lipase were screened using AUF-MS in both positive- and negative-ion modes. Based on the extracted ion chromatograms (Figure 2 and Figure 3), several compounds showing relatively high BD values in the presence of α-glucosidase and lipase were identified, and the corresponding BD results are presented in Table 3 and Table 4. These results indicate that ZZD contains multiple components showing preferential retention in the presence of α-glucosidase and lipase, providing candidate compounds for further investigation of their lipid-regulating activities.

In this study, multiple compounds in ZZD showing relatively high BD values toward lipase were identified (Table 3 and Table 4). Under negative-ion detection conditions, several compounds exhibited notable BD values, including naringin (49.69%), hesperidin (48.52%), nomilinic acid 17-O-beta-D-glucoside (54.31%), poncirin (62.20%), luteolin (23.99%), hesperetin (51.98%), and dicaffeoylquinic acid (approximately 59.49%). In the positive-ion mode, several other compounds also showed relatively high BD values, including gardenin B (48.81%) and artemetin (approximately 56%). Tangeretin showed a lower BD value of approximately 35.94%, while nobiletin and isosinensetin showed BD values of approximately 49% and 30%, respectively. Overall, at least 12 chemical constituents with relatively high BD values toward lipase were detected in ZZD, representing several structural classes, including flavonoids, limonoids, and organic acids.

We screened several compounds in ZZD that showed preferential retention in the presence of α-glucosidase. In the negative-ion mode, naringin, hesperidin, and poncirin showed relatively higher BD values, with binding rates of 19.49%, 19.85%, and 23.80%, respectively. In the positive-ion mode, stachydrine and gardenin B exhibited relatively lower BD values, with values of 8.19% and 5.90%, respectively. These findings indicate that naringin, poncirin and stachydrine showed detectable BD values toward α-glucosidase, although all BD values were below 30%.

In the affinity ultrafiltration screening against lipase, 12 compounds in ZZD exhibited relatively high BD values. Separately, in the α-glucosidase screening system, five compounds demonstrated detectable BD values, all with BD values below 30%. In addition, several compounds, including naringin, hesperidin, and poncirin, showed detectable BD values, suggesting that these compounds were retained under both screening conditions and warranting further investigation. The representative MS/MS spectra of three major flavonoids, including naringin, hesperidin, and poncirin, are provided in the Supplementary Materials (Figures S1–S3).

### 3.4. Cellular Effects of Naringin, Hesperidin, and Poncirin on FFA-Induced Lipid Accumulation

Based on the preferential retention of compounds in the affinity ultrafiltration assays, we found that several compounds exhibited high BD values to lipase, and the BD values of most active ingredients listed in Table 3 and Table 4. Among these compounds, we selected three—naringin, hesperidin, and poncirin—that exhibited BD values exceeding 30% and are commercially available to validate their preliminary cellular effects. To verify this hypothesis, we further investigated these three active compounds using an FFA-induced lipid deposition model in HepG2 cells. Indicators such as Oil Red O staining, Glu, and TG were measured to evaluate their potential lipid-related effects.

In HepG2 cells exposed to gradient concentrations of these three compounds, naringin showed no significant effect on cell viability, whereas hesperidin and poncirin caused concentration-dependent reductions in cell viability (Figure 4). Hesperidin at 62.5 μg/mL reduced cell viability to below 90%, while poncirin exhibited a more pronounced effect; even at 7.81 μg/mL, it significantly reduced cell viability (*p* ≤ 0.05).

As shown in Figure 5A, FFA successfully induced lipid accumulation in HepG2 cells, as evidenced by Oil Red O staining, which revealed clear lipid droplet deposition in the FFA group, supporting the establishment of the lipid deposition model. Positive control orlistat (250 μg/mL) significantly reduced lipid accumulation (*p* ≤ 0.05). Naringin was associated with reduced lipid deposition at all tested concentrations (Figure 5B, *p* ≤ 0.05). Furthermore, naringin at concentrations of 500 μg/mL and 250 μg/mL significantly reduced TG content in HepG2 cells (Figure 5C, *p* < 0.05), whereas no significant reduction in glucose levels was observed (Figure 5D).

Figure 6A reflects the effect of hesperidin on FFA-induced lipid deposition in HepG2 cells. The results showed that hesperidin at concentrations of 12.5, 25, and 50 μg/mL was associated with a significant reduction in lipid accumulation (Figure 6B). Further experiments demonstrated that hesperidin at 50 μg/mL as well as at lower concentrations like 25 and even down to 12.5 μg/mL significantly reduced FFA-induced TG content within HepG2 cells (Figure 6C; *p* < 0.05). Positive control orlistat (25 μg/mL) significantly reduced fat accumulation (*p* ≤ 0.05). Hesperidin treatment at 50, 25, and 12.5 μg/mL was associated with lower glucose levels; however, these changes were not statistically significant (Figure 6D). Taken together, these findings provide preliminary evidence that a potential lipid-related effect of hesperidin under the experimental conditions tested, supporting further investigation in future studies.

Poncirin was associated with a concentration-dependent reduction in lipid deposition in HepG2 cells, with a reduction observed at all tested concentrations (2.5–10 μg/mL), as shown in Figure 7A,B. Notably, poncirin at 2.5 μg/mL reduced lipid accumulation under the experimental conditions tested, while 10 μg/mL orlistat was included as a reference treatment (Figure 7A,B). At the tested concentrations of 10, 5, and 2.5 μg/mL, poncirin was associated with a significant reduction in cellular TG content (Figure 7, *p* < 0.05). Positive control orlistat (10 μg/mL) significantly reduced fat accumulation (*p* ≤ 0.05); however, under the same experimental conditions, no significant change was observed in the glucose level (Figure 7D, *p* > 0.05).

## 4. Discussion

ZZD is a classic TCM formula, and it is traditionally considered to have the function of “fortifying the spleen and resolving turbidity,” which has been regarded as being related to the regulation of lipid metabolism. Previous animal studies have shown that ZZD, as well as its individual herbal components, may have the ability to reduce blood lipid levels [7,8,9]. HepG2 cells are a commonly used human hepatoma cell line, and they retain important metabolic functions of hepatocytes. These cells are also widely used to study the direct effects of drugs on glucose and lipid metabolism [20]. Therefore, HepG2 cells were used in this study to further investigate the potential effects of ZZD on glucose and lipid metabolism in hepatocytes.

The effects of ZZD on lipid metabolism showed a dose-dependent trend, although the most effective concentration varied across different indicators. For glucose, the medium concentration (250 μg/mL) produced the most obvious reduction. In contrast, the higher concentration (500 μg/mL) showed the strongest effect on reducing both TG and total T-CHO, indicating that different metabolic indicators responded differently to the treatment concentration. This reflects the compound complexity of ZZD, which may elicit distinct optimal concentration responses across different metabolic pathways, or even trigger complex feedback regulation at higher doses.

In the process of screening for the active ingredients in ZZD, we primarily used bioaffinity ultrafiltration. This method is based primarily on the action of a molecular weight-selective membrane. Ultrafiltration membranes retain enzyme-ligand complexes and remove unretained compounds. This mechanism helps enrich potential active ingredients and improve the efficiency of compound screening. We then used UPLC-Q-TOF-MS/MS technology to further analyze the retained compounds. This process allows us to identify compounds that show preferential retention in the presence of the target enzyme. α-Glucosidase and lipase play important roles in glucose and lipid metabolism in the human body [21,22]. Using them simultaneously as target enzymes not only enables the screening of lipid-lowering active compounds but may also lead to the unexpected discovery of glucose-lowering active compounds. α-Glucosidase plays a central role in regulating postprandial blood glucose [23]. Under pathological conditions, its activity is excessively elevated. This leads to rapid carbohydrate digestion and absorption, often resulting in a significant increase in postprandial blood glucose [24]. If α-glucosidase activity is too high, it may lead to elevated blood glucose levels [23,24]. Therefore, active compounds capable of inhibiting α-glucosidase may serve as antidiabetic potential candidates [24]. Since glucose metabolism and lipid metabolism in the body are inextricably linked, this further affects lipid metabolism. Compounds that inhibit lipase also have potential as drugs for treating obesity [25]. Their simultaneous use provides a practical strategy for screening compounds potentially related to both glucose- and lipid-metabolism regulation from the complex ZZD extract. However, the preferential retention of compounds in the presence of these enzymes does not necessarily indicate a direct mechanism underlying intracellular lipid regulation. In the present study, lipase screening was mainly used as a target-based screening criterion, while the subsequent FFA-induced HepG2 model was used for functional validation. The observed reductions in lipid accumulation and TG levels therefore provide complementary evidence of cellular lipid-regulating activity, rather than direct evidence that these effects are mediated by lipase inhibition. Other metabolic targets and signaling pathways may also contribute to the observed effects of ZZD, but their systematic investigation was beyond the scope of the present study.

Based on the results of affinity ultrafiltration, we found that at least 12 compounds showed relatively high BD values in the presence of lipase, including flavonoids, limonoids, and alkaloids. Several compounds showed preferential retention in the presence of the target enzymes and exhibited lipid-regulating effects in the cell model. These findings suggest that multiple constituents of ZZD may contribute to its biological activity. Some compounds, like poncirin, were found in both positive and negative-ion modes, and showed relatively high BD values in both analyses. This observation provides additional preliminary support for poncirin as a putative candidate identified by the AUF-MS workflow. Polymethoxyflavones like nobiletin and tangeretin basically showed moderate or relatively strong BD values under the positive ion mode. This might mean that the methoxy groups in their structures may be related to their biological activity, though it is not absolutely certain. These findings provide further evidence for the presence of multiple compounds showing relatively high BD values in the presence of lipase in ZZD and offer a basis for further investigation of their potential roles in lipid metabolism.

Naringin, hesperidin, and poncirin are three major active flavonoids commonly found in citrus-derived TCM such as *Citrus aurantium* (Zhishi). Previous pharmacological studies have shown that *Citrus aurantium* has several biological activities, including antioxidant [26], anti-inflammatory [27], and lipid-regulating effects [28]. These activities are probably related to the different flavonoids present in this herb. Earlier studies have also reported that flavonoids possess a wide range of biological functions, and many of these effects are associated with their antioxidant and anti-inflammatory properties [27,29]. According to published reports, naringin [30,31,32,33], hesperidin [34], and poncirin [35,36] all exhibit obvious antioxidant and anti-inflammatory activities. In addition, naringin [37] and poncirin [38] have also been reported to show anti-tumor effects. Concerning glucose and lipid metabolism, the present study found that naringin was associated with reduced lipid accumulation in the FFA-induced HepG2 cell model under the tested conditions. A reduction in lipid accumulation was also observed at concentrations as low as 125 μg/mL. These findings provide preliminary evidence supporting further investigation of naringin as a candidate compound associated with lipid-regulating activity. This compound has previously been reported to have a protective effect against fatty liver disease, which is consistent with the findings of our study [39].

Poncirin, naringin, and hesperidin were all associated with significant reductions in lipid accumulation under the tested conditions, but poncirin demonstrated an advantage at lower doses. Among them, poncirin demonstrated the advantage of lower dosages, but also showed a concentration-dependent reduction in cell viability. It inhibited cell proliferation even at concentrations as low as 7.81 μg/mL. Therefore, the contribution of changes in cellular metabolic activity to the observed lipid reduction cannot be fully excluded, particularly when interpreting the effects of poncirin at higher concentrations. Its effective concentration range may be a key factor to consider when evaluating its potential for lipid-regulating applications. Another compound, hesperidin, showed lipid-regulating effects across the tested concentration range. At 12.5 μg/mL, hesperidin reduced lipid accumulation under the experimental conditions tested. These results indicate that hesperidin offers a better balance between efficacy and effective concentration range. Although poncirin and hesperidin are structurally similar and both belong to the flavonoid class, they exhibit different biological effects and effective concentration ranges, indicating that even minor structural differences in compounds can have a significant impact on their biological activity. Based on the above results, hesperidin exhibits a better balance of efficacy and effective concentration range compared to the other two compounds. This suggests that hesperidin may be more suitable as a potential lipid-regulating compound or functional food ingredient. Future studies need to dig deeper into its molecular mechanism and also confirm the lipid-lowering effects by through in vivo experiments. Currently, poncirin is known to act directly on various tumor cells by regulating intracellular signaling pathways and inducing mitochondria-mediated apoptosis [38,40]. This suggests that, in addition to its lipid-lowering effects, poncirin also has the potential to serve as an anticancer drug. However, its oral bioavailability, metabolic profile in vivo, and toxicity still require more systematic investigation.

Several limitations of this study should be noted. First, while AUF-MS screening and HepG2 models provided preliminary evidence for the preferential retention of selected candidates and their lipid-related effects, downstream mechanisms (such as AMPK pathway activation) and in vivo efficacy remain to be validated in animal models, particularly given pharmacokinetic constraints like low oral bioavailability and active metabolites. Second, AUF experiments were performed as single measurements without membrane-related controls or evaluations of recovery and nonspecific adsorption, making BD values preliminary and preventing the complete exclusion of nonspecific retention. In addition, the potential contribution of membrane–analyte interactions and membrane fouling to compound retention was not independently evaluated in this study. Therefore, the observed retention should be interpreted as preferential enrichment in the AUF-MS workflow rather than definitive evidence of direct enzyme binding. Finally, cell viability was evaluated in complete medium rather than under FFA-treatment conditions; for poncirin, which reduced CCK-8 activity at functional concentrations, the contribution of altered cellular metabolic activity to lipid reduction cannot be fully ruled out.

## 5. Conclusions

This study developed a membrane-based AUF-MS approach for the preliminary screening of candidate candidates from the TCM formula ZZD. This method utilizes the selective retention properties of ultrafiltration membranes to enrich compounds showing preferential retention in the presence of the target enzymes, α-glucosidase and lipase. These compounds are then further analyzed and putatively identified using UPLC-Q-TOF-MS/MS. Our screening results identified multiple candidate compounds in ZZD showing relatively high BD values in the presence of lipase, among which naringin, hesperidin, and poncirin exhibited relatively higher BD values than other identified components. In addition, several compounds, including naringin and poncirin, demonstrated detectable BD values toward α-glucosidase. Further in vitro validation studies showed that naringin and hesperidin exhibited an effective concentration range, while poncirin was associated with reduced lipid accumulation in HepG2 cells under the tested conditions. However, the possibility of an influence of altered cellular viability or metabolic activity under FFA conditions cannot be completely excluded. Taken together, these findings provide preliminary evidence supporting the utility of the membrane-based, multi-target AUF-MS method for screening candidate compounds showing preferential retention in the presence of the target enzymes in ZZD. This approach may also be applicable to the preliminary screening of putative bioactive compounds from other natural medicines. Overall, naringin and hesperidin may serve as potential candidates for further investigation of their lipid-regulating activities.

## Figures and Tables

**Figure 1:**
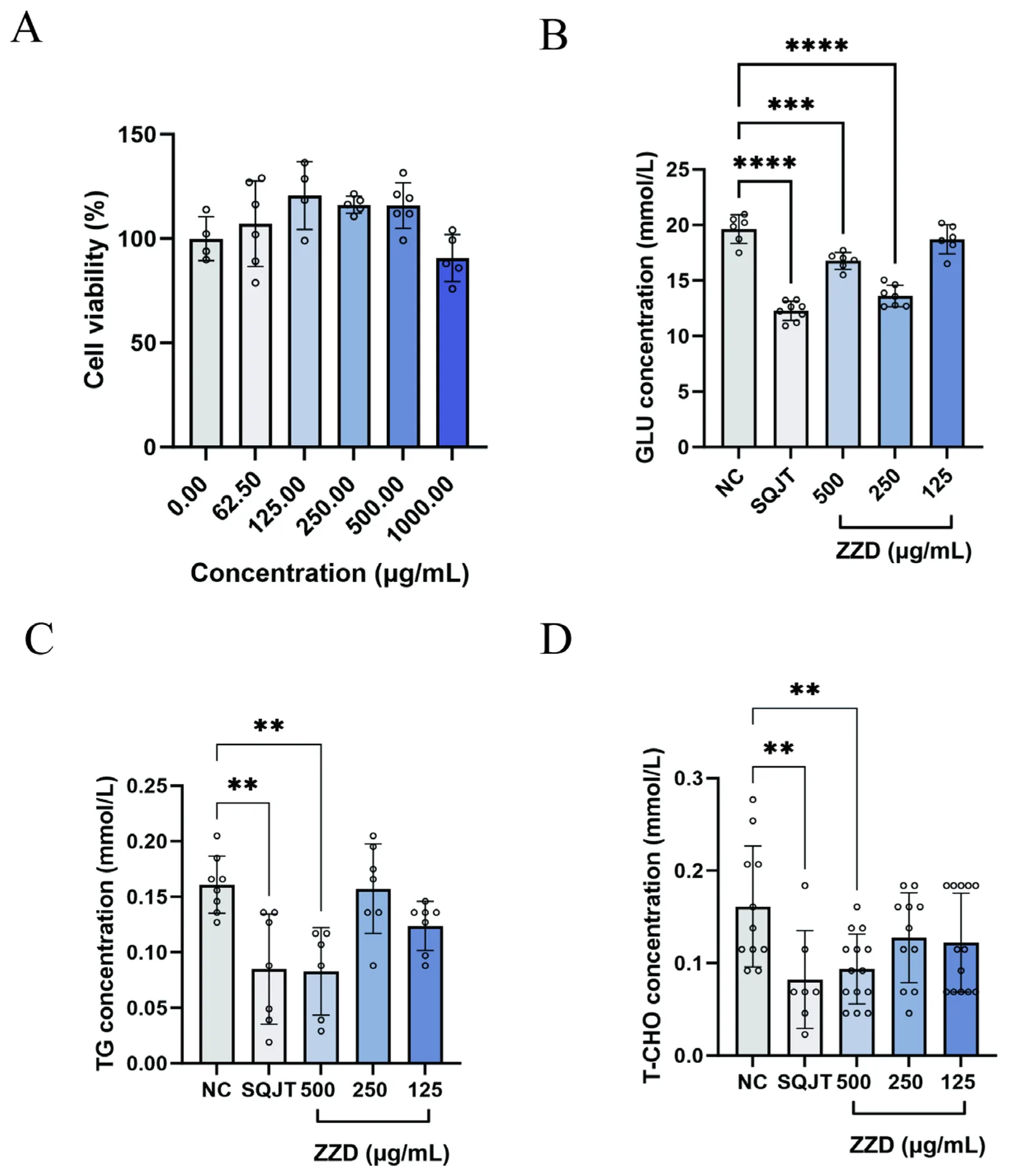
Effect of Zhizhu Decoction on glucose and lipid metabolism in HepG2 cells. (**A**) cell viability (*n* = 4–6 per group), (**B**) GLU, glucose (*n* = 6–8 per group), (**C**) TG, triglycerides (*n* = 6–8 per group), (**D**) T-CHO, Total cholesterol (*n* = 7–14 per group). Data are presented as mean ± SD of independent biological replicates. **** *p* < 0.0001, *** *p* < 0.001, ** *p* < 0.01 vs. the NC (control) group.

**Figure 2:**
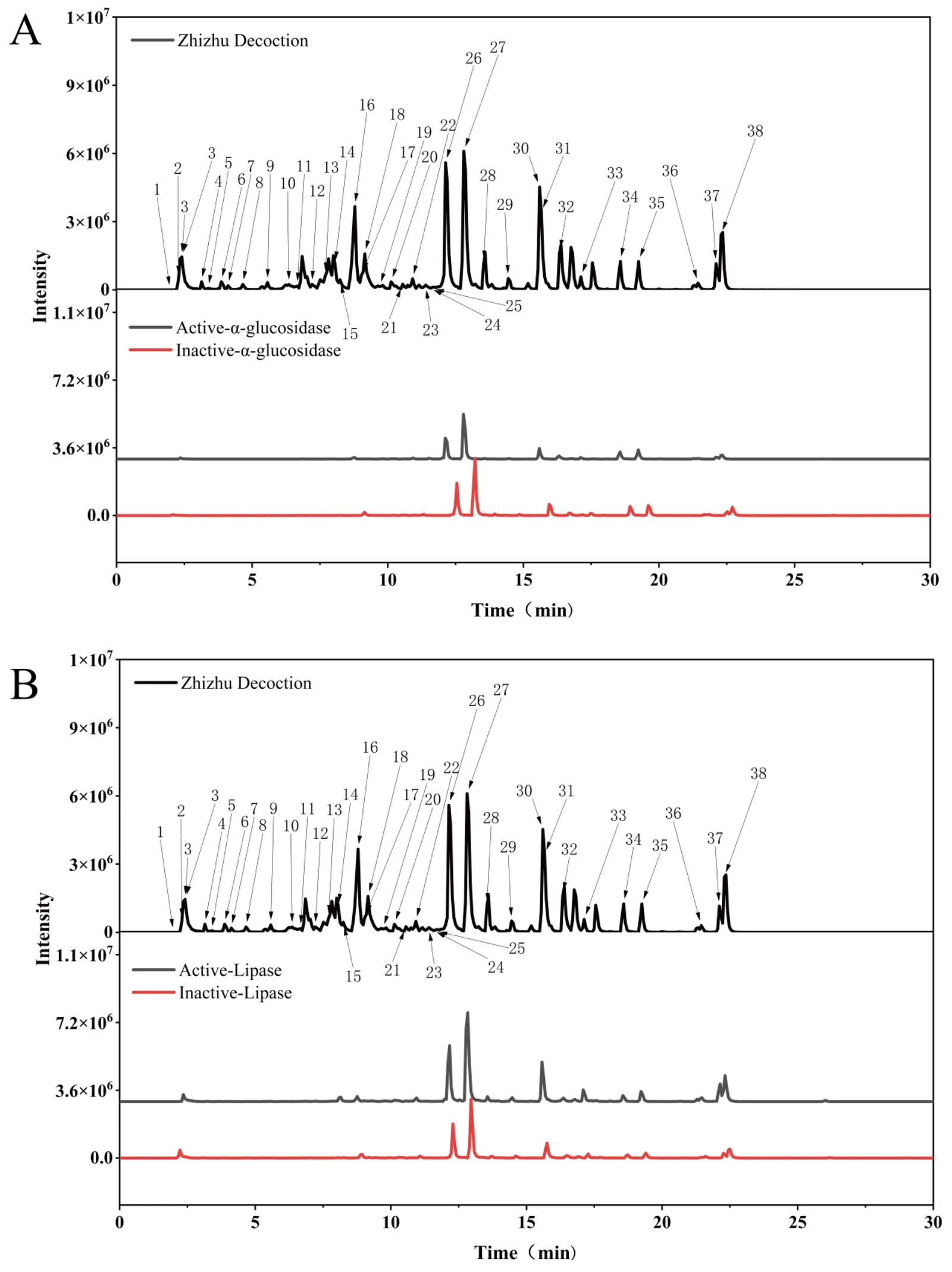
Affinity ultrafiltration results of Zhizhu Decoction in negative-ion mode with (**A**) α-glucosidase and (**B**) lipase. The numbers (1–38) denote the major peaks identified in the decoction in negative-ion mode.

**Figure 3:**
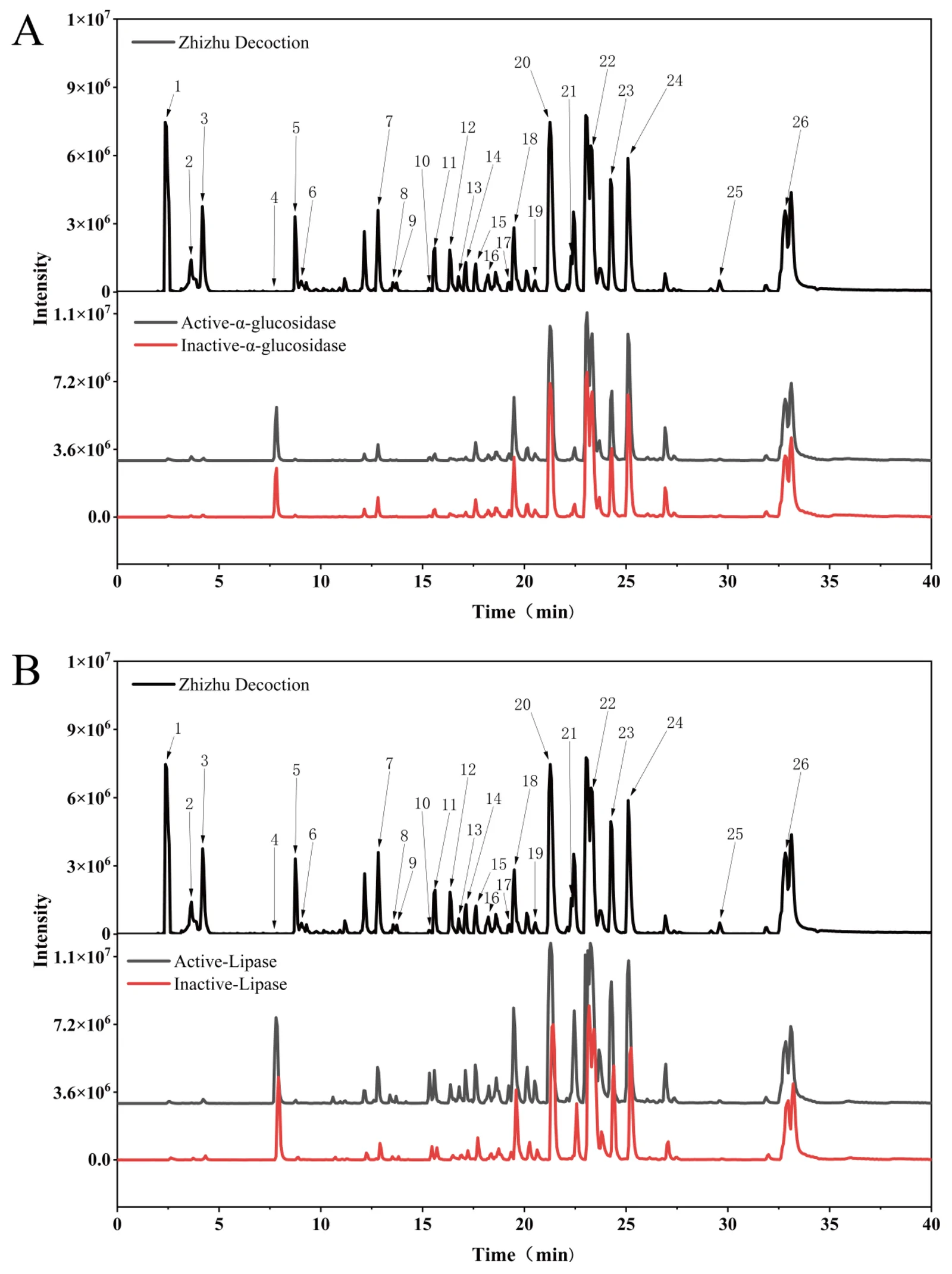
Affinity ultrafiltration results of Zhizhu Decoction in positive ion mode with (**A**) α-glucosidase and (**B**) lipase. The numbers (1–26) denote the major peaks identified in the decoction in positive-ion mode.

**Figure 4:**
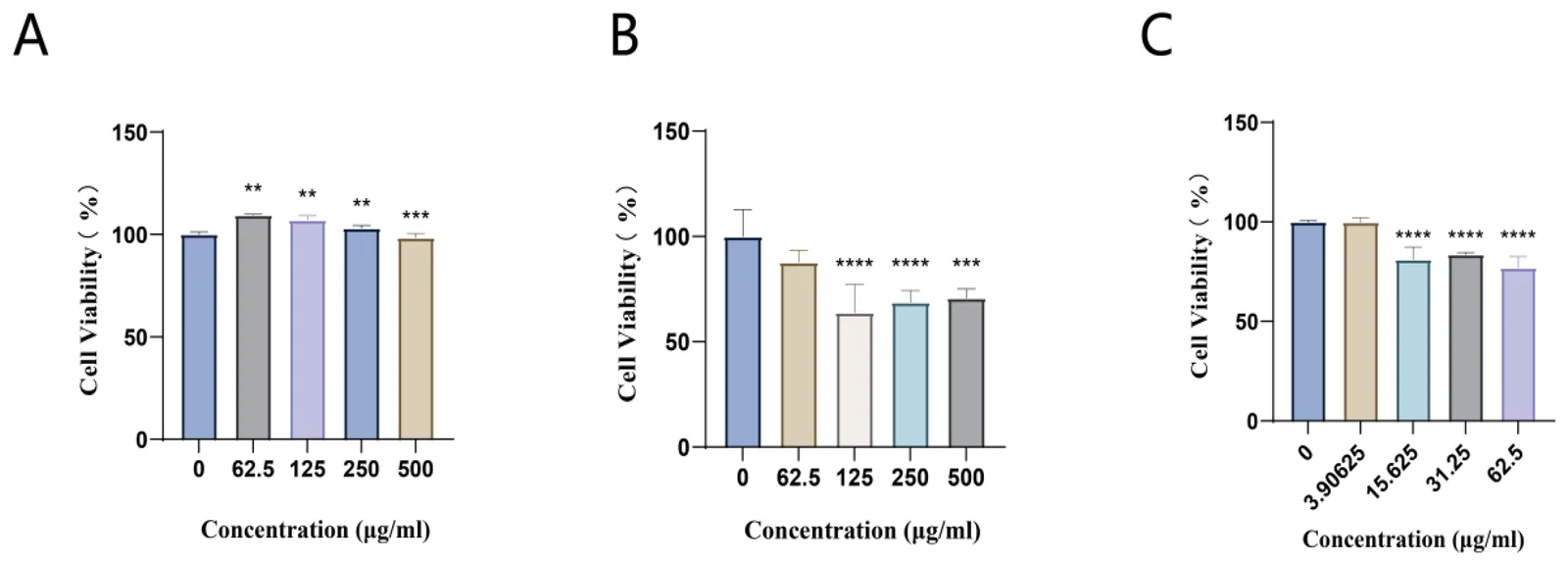
Effect of (**A**) naringin (*n* = 4–6 per group), (**B**) hesperidin (*n* = 4–6 per group), and (**C**) poncirin (*n* = 4–6 per group) on cell viability. Data are presented as mean ± SD of independent biological replicates. **** *p* < 0.0001, *** *p* < 0.001, ** *p* < 0.01 vs. the 0 group.

**Figure 5:**
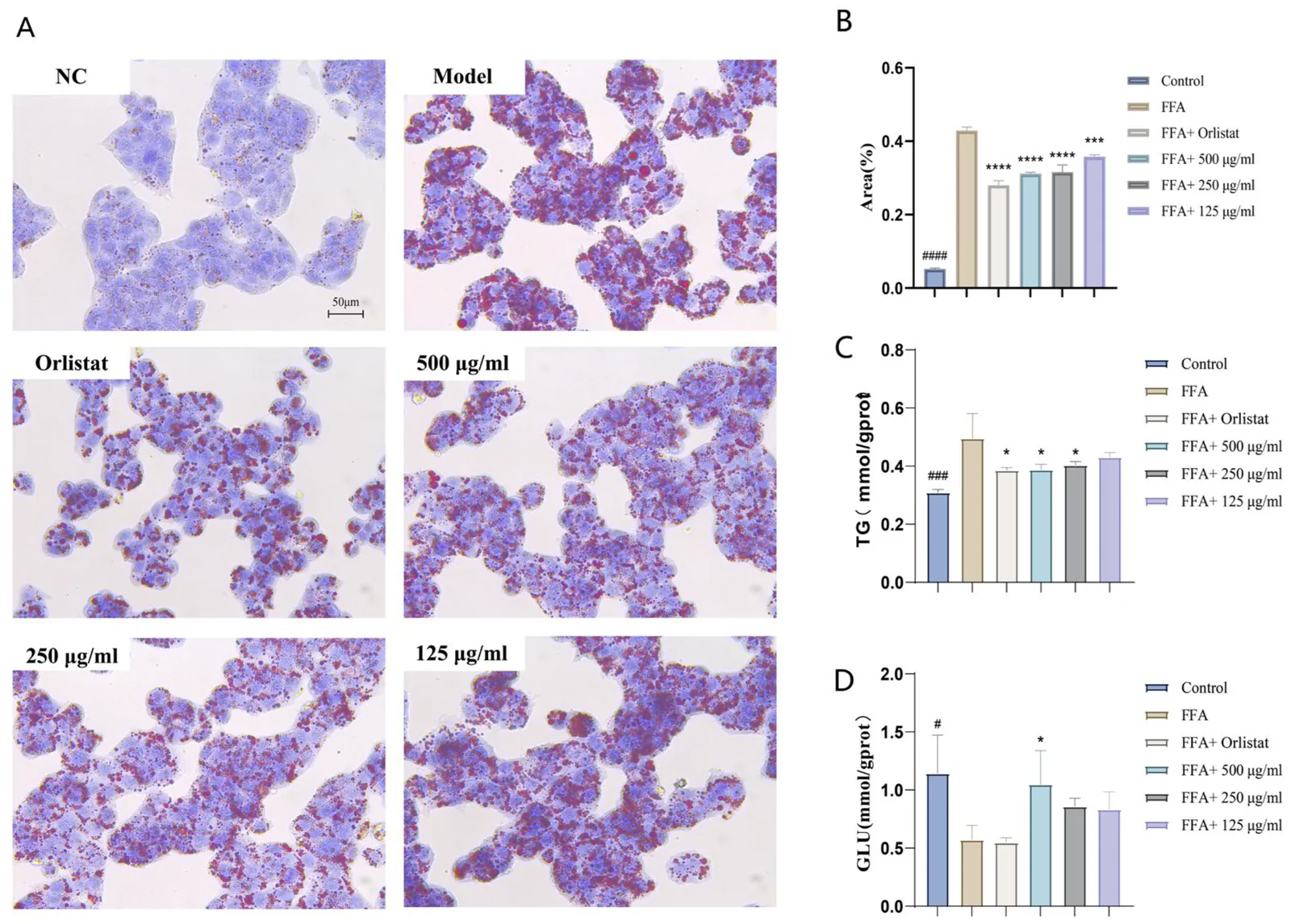
Effect of naringin on FFA-induced lipid accumulation in HepG2 cells. (**A**) Oil red O staining. (**B**) Quantification of the Oil red O-stained area. (**C**) Triglyceride concentration. (**D**) Glucose concentration. Data are presented as mean ± SD of independent biological replicates, *n* = 3 per group. **** *p* < 0.0001, *** *p* < 0.001, * *p* < 0.05 vs. the FFA model group, ^####^ *p* < 0.0001, ^###^ *p* < 0.001, ^#^ *p* < 0.05 vs. the FFA model group.

**Figure 6:**
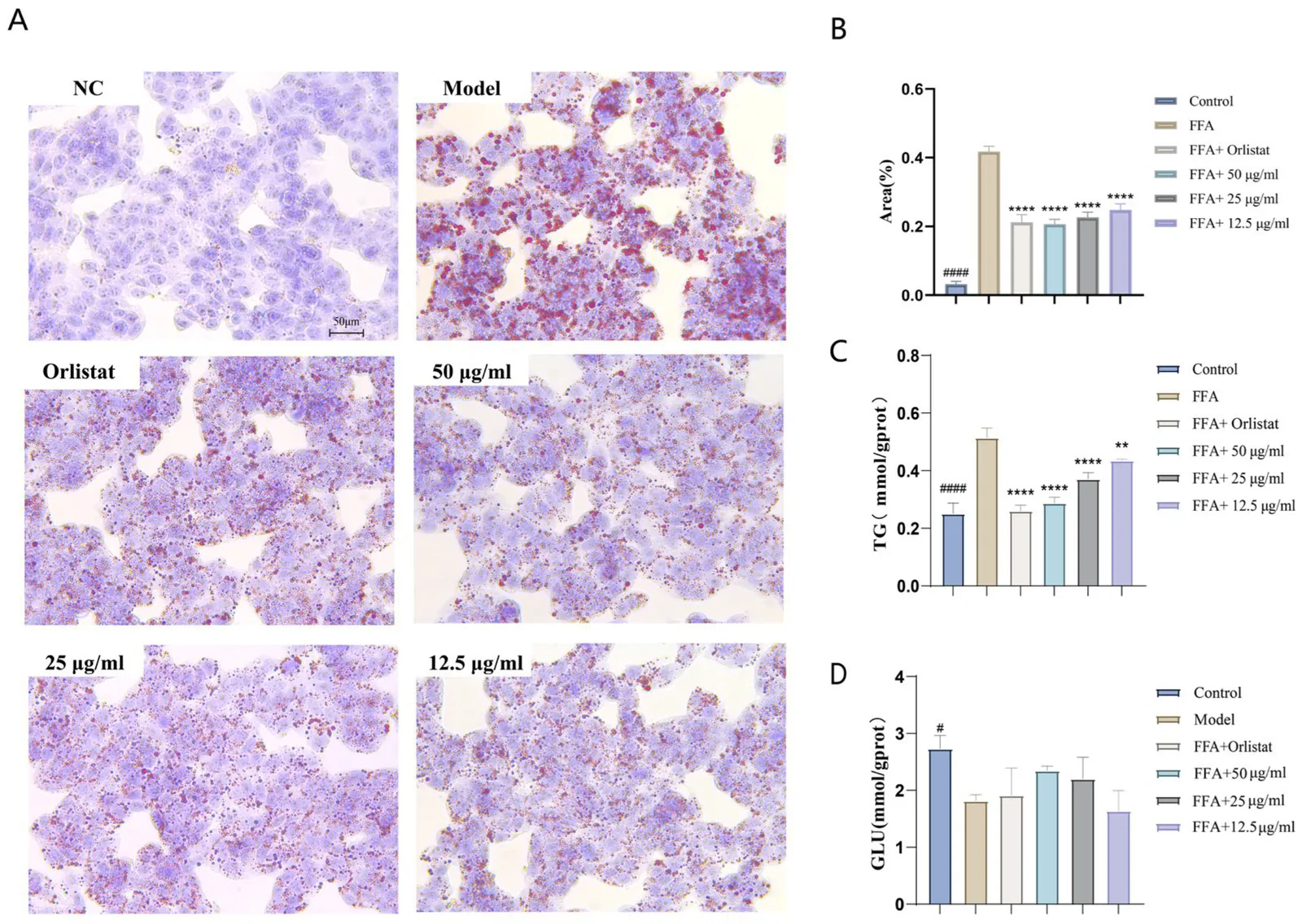
Effect of hesperidin on FFA-induced lipid accumulation in HepG2 cells. (**A**) Oil red O staining. (**B**) Quantification of the Oil red O-stained area. (**C**) Triglyceride concentration. (**D**) Glucose concentration. Data are presented as mean ± SD of independent biological replicates, *n* = 3 per group. **** *p* < 0.0001, ** *p* < 0.005 vs. the FFA (model) group, ^####^ *p* < 0.0001, ^#^ *p* < 0.05 vs. the FFA (model) group.

**Figure 7:**
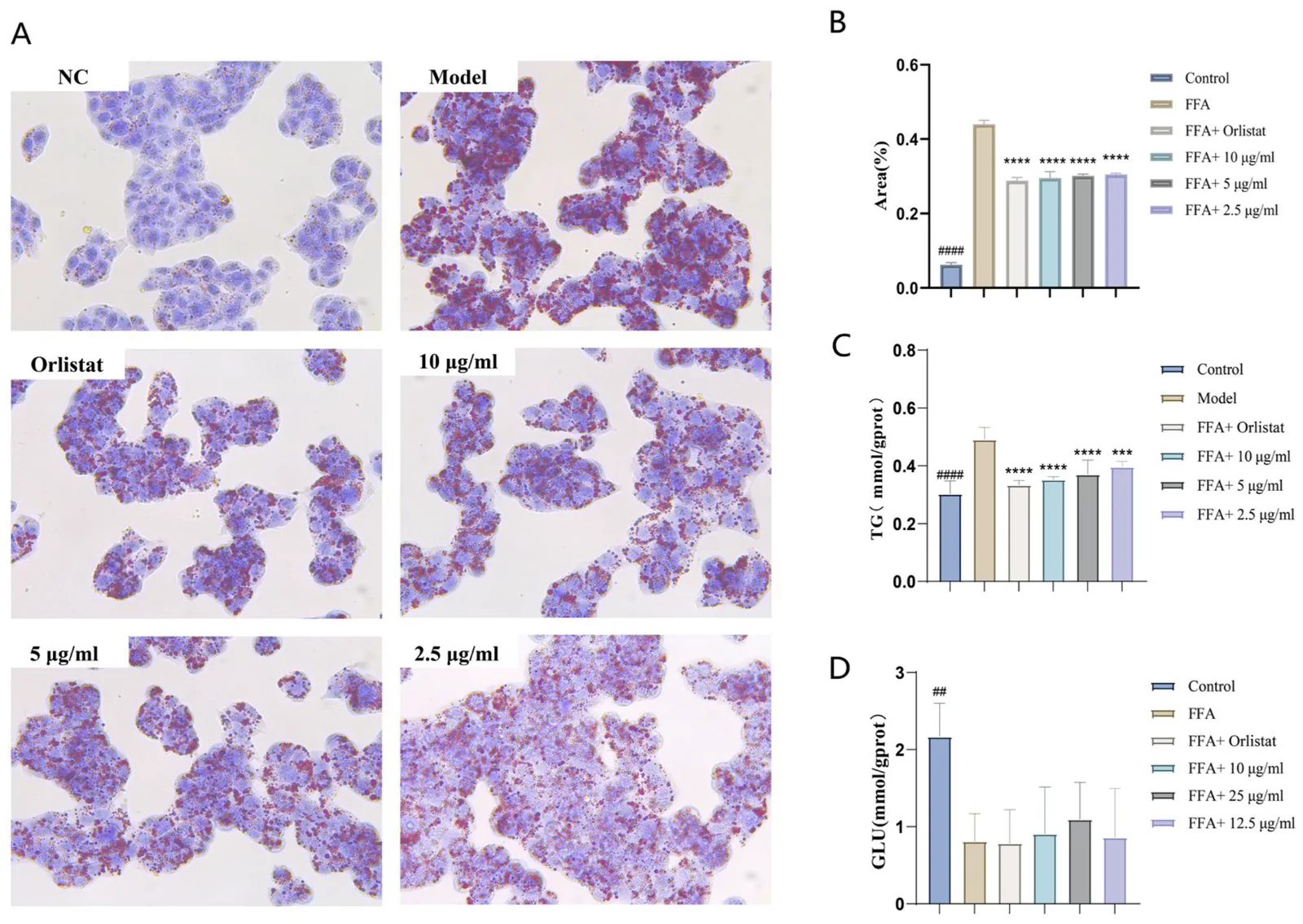
Effect of poncirin on FFA-induced lipid accumulation in HepG2 cells. (**A**) Oil red O staining. (**B**) Quantification of the Oil red O-stained area, *n* = 3 per group. (**C**) Triglyceride concentration, *n* = 5 per group. (**D**) Glucose concentration, *n* = 3 per group. Data are presented as mean ± SD of independent biological replicates. **** *p* < 0.0001, *** *p* < 0.001 vs. the FFA (model) group, ^####^ *p* < 0.0001, ^##^ *p* < 0.005 vs. the FFA (model) group.

**Table 1:** Putative identification of compounds in Zhizhu Decoction extract (negative-ion mode).

Peak	RT	Putative Compound	Ion/Adduct	Theoretical *m*/*z*	Measured *m*/*z*	Error (ppm)	Molecular Formula	MS/MS (*m*/*z*)	Source/Reference
1	1.987	Unidentified	-	-	158.9704	-	-	141.898, 159.2541	
2	2.291	Galactaric Acid	[M−H]−	209.0303	209.0303	0.00	C_6_H_10_O_8_	209.0298, 191.0191, 173.0088	HMDB0000639
3	2.413	D-(-)-Quinic acid	[M−H]−	191.0556	191.0560	−2.09	C_7_H_12_O_6_	109.0288, 127.0394, 191.0557	Agilent PCDL
4	3.143	1F-fructofuranosylnystose	[M−H]−	827.2674	827.2673	0.12	C_30_H_52_O_26_	179.0558, 161.0456, 341.1084	Agilent PCDL
5	3.386	Hesperidin isomer	[M−H]−	609.1825	609.1820	0.82	C_28_H_34_O_15_	125.0240, 373.0758	
6	3.872	Unidentified	-	-	779.2463	-	-	719.2232, 779.2454	
7	4.115	Unidentified	-	-	645.1879	-	-	645.1869	
8	4.662	Unidentified	-	-	359.0619	-	-	167.0346, 315.0709, 359.0618	
9	5.574	Unidentified	-	-	315.0721	-	-	315.0714, 152.0106, 108.0209	
10	6.364	Unidentified	-	-	291.0725	-	-	129.0188	
11	6.668	Vanillic acid	[M−H]−	167.0349	167.0349	0.00	C_8_H_8_O_4_	167.0346	HMDB0000484
12	7.215	Cichoriin	[M−H]−	339.0721	339.0717	1.18	C_15_H_16_O_9_	177.0188, 133.0290	HMDB0030821
13	7.701	2-trans-O-Feruloylglucaric acid	[M−H]−	385.0776	385.0777	−0.26	C_16_H_18_O_11_	147.0296, 191.0193, 313.0566	HMDB0302546
14	8.047	Unidentified	-	-	357.2134	-	-	275.5302, 313.2235, 357.2133	
15	8.249	Caffeoyl C1-glucuronide	[M−H]−	355.0671	355.0675	−1.13	C_15_H_16_O_10_	147.0290, 355.0694	HMDB0041709
16	8.796	Lonicerin	[M−H]−	593.1511	593.1509	0.34	C_27_H_30_O_15_	593.1510	[17]
17	9.039	Narcissin	[M−H]−	623.1623	623.1620	0.48	C_28_H_32_O_16_	623.1608	[18,19]
18	9.161	Unidentified	-	-	313.0565	-	-	283.0235, 313.0712	
19	9.708	Caffeoylmalic acid	[M−H]−	295.0459	295.0454	1.69	C_13_H_12_O_8_	121.0285, 135.0447, 181.9238, 251.0547	HMDB0029318
20	10.15	Unidentified	-	-	771.2346	-	-	301.0709, 325.0702, 463.1236, 487.1240, 609.1812	
21	10.559	Vicenin III	[M−H]−	563.1406	563.1409	−0.53	C_26_H_28_O_14_	149.0440, 293.0450, 323.0559, 341.0665, 413.0872	Agilent PCDL
22	10.924	Neoeriocitrin	[M−H]−	595.1668	595.1673	−0.84	C_27_H_32_O_15_	287.0588	HMDB0255521
23	11.41	p-Coumaric acid	[M−H]−	163.0395	163.0389	3.68	C_9_H_8_O_3_	119.0497	Agilent PCDL
24	11.532	Unidentified	-	-	609.2546	-	-	161.0451, 307.1911, 609.1825	
25	11.575	Unidentified	-	-	447.0918	-	-	357.0611	
26	12.14	Naringin	[M−H]−	579.1710	579.1760	−8.63	C_27_H_32_O_14_	271.0616	HMDB0002927, [18]
27	12.942	Hesperidin	[M−H]−	609.1815	609.1822	−1.15	C_28_H_34_O_15_	301.0717, 609.1820	HMDB0003265
28	13.539	Nomilinic acid 17-O-beta-D-glucoside	[M−H]−	711.2864	711.2873	−1.27	C_34_H_48_O_16_	607.2750, 651.2648	HMDB0302541
29	14.451	Obacunone 17-O-beta-D-glucoside	[M−H]−	633.2553	633.2555	−0.32	C_32_H_42_O_13_	161.0448, 589.2636	HMDB0302542
30	15.539	Poncirin	[M−H]−	593.1869	593.1864	0.84	C_28_H_34_O_14_	285.0768	HMDB0037487, [18]
31	15.667	Kaempferol 3-O-α-L-arabinopyranoside	[M−H]−	417.0827	417.0834	−1.68	C_21_H_22_O_9_	417.0822	HMDB0029502
32	16.318	Luteolin	[M−H]−	285.0404	285.0395	3.16	C_15_H_10_O_6_	133.0287, 285.0393	HMDB0005800
33	17.126	Natsudaidain 3-(4-O-3-hydroxy-3-methylglutaroylglucoside)	[M−H]−	723.2186	723.2134	7.19	C_33_H_40_O_18_	417.1187, 723.2126	HMDB0040812
34	18.586	Pinobanksin	[M−H]−	271.0612	271.0611	0.37	C_15_H_12_O_5_	119.0495, 151.0026, 177.0177, 271.0593	Agilent PCDL
35	19.254	Hesperetin	[M−H]−	301.0707	301.0718	−3.65	C_16_H_14_O_6_	107.0131, 108.0208, 134.0364, 151.0031, 177.0178	HMDB0005782, [18]
36	21.443	Unidentified	-	-	517.2088	-	-	355.0821, 399.0717	
37	22.111	Unidentified	-	-	531.2237	-	-	531.2228	
38	22.294	Dicaffeoylquinic acid	[M−H]−	515.1194	515.1176	3.49	C_25_H_24_O_12_	121.0293, 349.1424, 469.1864	HMDB0029280

**Table 2:** Putative identification of compounds in Zhizhu Decoction extract (positive ion mode).

Peak	RT	Putative Compound	Ion/Adduct	Theoretical *m*/*z*	Measured *m*/*z*	Error (ppm)	Molecular Formula	MS/MS (*m*/*z*)	Source/Reference
1	2.367	Stachydrine	[M+H]+	144.1019	144.1016	2.08	C_7_H_13_NO_2_	102.0543, 144.1023	Agilent PCDL
2	3.643	Adenosine	[M+H]+	268.1039	268.1030	3.36	C_10_H_13_N_5_O_4_	136.0611, 268.1028	HMDB0000050
3	4.19	Phenylacetaldehyde	[M+H]+	121.0648	121.0649	−0.83	C_8_H_8_O	103.0535, 121.0637	HMDB0006236
4	7.719	Unidentified	-	-	344.2256	-	-	133.0846, 195.1206, 327.1986, 344.2249	
5	8.747	Lonicerin	[M+H]+	595.1663	595.1650	2.18	C_27_H_30_O_15_	593.1510	[17]
6	9.051	Unidentified	-	-	625.1743	-	-	623.1608	
7	12.82	Unidentified	-	-	611.1964	-	-	129.0540, 147.0643, 177.0532, 303.0851	
8	13.549	Nomilinic acid	[M+H]+	533.2381	533.2363	3.38	C_28_H_36_O_10_	455.2043, 487.2299, 515.2250, 533.2350	HMDB0035771
9	13.732	Unidentified	-	-	521.1638	-	-	137.0575, 359.1100, 461.0828, 521.1623	
10	15.337	Unidentified	-	-	507.1471	-	-	345.0945, 507.1471	
11	15.556	Poncirin	[M+H]+	595.2022	595.2001	3.53	C_28_H_34_O_14_	285.0768	HMDB0037487, [18]
12	16.346	Unidentified	-	-	728.3957	-	-	728.3952	
13	16.771	Unidentified	-	-	581.1845	-	-	404.1074, 419.1320	
14	17.136	Natsudaidain 3-(4-O-3-hydroxy-3-methylglutaroylglucoside)	[M+H]+	725.2287	725.2262	3.45	C_33_H_40_O_18_	419.1321, 725.2257	HMDB0040812
15	17.623	Gardenin B	[M+H]+	359.1125	359.1113	3.34	C_19_H_18_O_7_	298.0807, 344.0873, 359.1109	HMDB0302154
16	18.23	Salvigenin	[M+H]+	329.1019	329.1007	3.65	C_18_H_16_O_6_	268.0711, 285.0737, 314.0763, 329.1004	HMDB0128577
17	19.264	Artemetin	[M+H]+	389.1231	389.1219	3.08	C_20_H_20_O_8_	197.0067, 359.0746, 359.0747	HMDB0030095
18	19.507	Tangeretin	[M+H]+	373.1281	373.1275	1.61	C_20_H_20_O_7_	153.0172, 287.0897, 315.0848, 343.0797	HMDB0030539, [18]
19	20.54	Nobiletin	[M+H]+	403.1387	403.1377	2.48	C_21_H_22_O_8_	137.0558, 315.0480, 373.0898	HMDB0029540, [18]
20	21.27	Sinensetin	[M+H]+	373.1271	373.1262	2.41	C_20_H_20_O_7_	315.0846, 343.0800, 357.0951	HMDB0036633, [18]
21	22.303	Limonin	[M+H]+	471.2013	471.1998	3.18	C_26_H_30_O_8_	161.0584, 425.1936, 453.1887	HMDB0035921, [18]
22	23.336	5,7,8,4′-Tetramethoxyflavone	[M+H]+	343.1176	343.1192	−4.66	C_19_H_18_O_6_	282.0871, 313.0694, 343.1161	Agilent PCDL
23	24.248	Unidentified	-	-	433.1499	-	-	403.0995, 433.1471	
24	25.099	Isosinensetin	[M+H]+	373.1281	373.1290	−2.41	C_20_H_20_O_7_	343.0789, 373.1265	HMDB0037599, [18]
25	29.598	Atractylenolide II	[M+H]+	233.1536	233.1528	3.43	C_15_H_20_O_2_	187.1470, 215.1420, 233.1521	HMDB0248707
26	32.821	Unidentified	-		163.0751			105.0328, 163.0744	

**Table 3:** Binding degree of active components in Zhizhu Decoction extract with α-glucosidase and lipase (negative-ion mode).

Peak	The Chemical Constituents	Binding Degree (%)	
		α-Glucosidase	Lipase
26	Naringin	19.49	49.69
27	Hesperidin	19.85	48.52
28	Nomilinic acid 17-O-beta-D-glucoside	-	54.31
30	Poncirin	23.80	62.20
32	Luteolin	-	23.99
35	Hesperetin	-	51.98
38	Dicaffeoylquinic acid	-	59.49

Note: “-” denotes not detected or values below 0.

**Table 4:** Binding degree of active components in Zhizhu Decoction extract with α-glucosidase and lipase (positive ion mode).

Peak	The Chemical Constituents	Binding Degree (%)	
		α-Glucosidase	Lipase
1	Stachydrine	8.19	-
15	Gardenin B	5.9	48.81
17	Artemetin	-	56.01
18	Tangeretin		35.94
19	Nobiletin	-	48.79
24	Isosinensetin	-	29.59

Note: “-” denotes not detected or values below 0.

## Data Availability

The raw data supporting the conclusions of this article will be made available by the authors on request.

## Supplementary Materials

The following supporting information can be downloaded at: https://www.mdpi.com/article/10.3390/membranes16100322/s1, Figure S1: RRepresentative MS/MS spectrum of naringin (negative ion mode); Figure S2: Representative MS/MS spectrum of hesperidin (negative ion mode); Figure S3: Representative MS/MS spectrum of poncirin (negative ion mode).

## Abbreviations

APAP	Acetaminophen
AUF-MS	Affinity Ultrafiltration-Mass Spectrometry
BSA	Bovine Serum Albumin
CCK-8	Cell Counting Kit-8
DMEM	Dulbecco’s Modified Eagle’s Medium
DMSO	Dimethyl Sulfoxide
FBS	Fetal Bovine Serum
FFA	Free Fatty Acid
GLU	Glucose
HDL-C	High-Density Lipoprotein Cholesterol
IDF	International Diabetes Federation
LDL-C	Low-Density Lipoprotein Cholesterol
MASLD	Metabolic Associated Steatotic Liver Disease
MWCO	Molecular Weight Cut-Off
OA	Oleic Acid/Sodium Oleate
PA	Palmitic Acid/Sodium Palmitate
PBS	Phosphate Buffered Saline
PUMA	p53 Up-regulated Modulator of Apoptosis
RCT	Reverse Cholesterol Transport
SQJT	Shenqi Jiangtang Granules
T-CHO	Total Cholesterol
TC	Total Cholesterol
TCM	Traditional Chinese Medicine
TG	Triglyceride
UPLC-QTOF-MS/MS	Ultra Performance Liquid Chromatography—Quadrupole Time-of-Flight Tandem Mass Spectrometry
ZZD	Zhizhu Decoction
