# Supplementary material for: Rapid Screening and Analysis of Putative Bioactive Candidates in Zhizhu Decoction Using Membrane-Based Affinity Ultrafiltration-Mass Spectrometry
Source: Membranes (Basel). 2026 Sep 27;16(10):322. doi: 10.3390/membranes16100322 (PMC13706955; doi:10.3390/membranes16100322)
Supplement: Supplementary file 1 [file membranes-16-00322-s001.zip › membranes-4460665-supplementary.pdf]

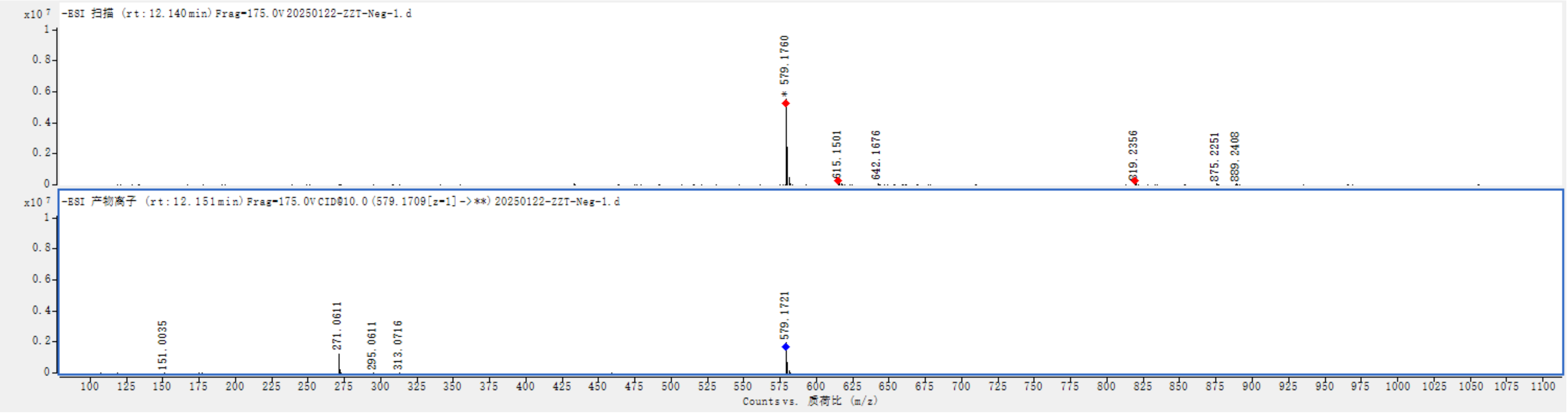

Supplementary Figure S1. Representative MS/MS spectrum of naringin (negative ion mode).

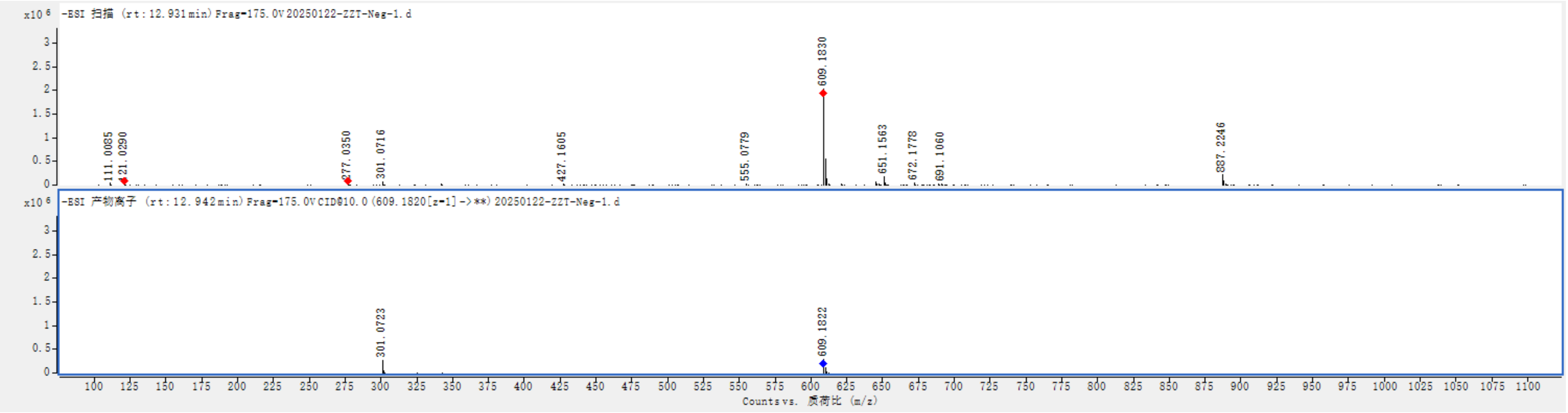

Supplementary Figure S2. Representative MS/MS spectrum of hesperidin (negative ion mode).

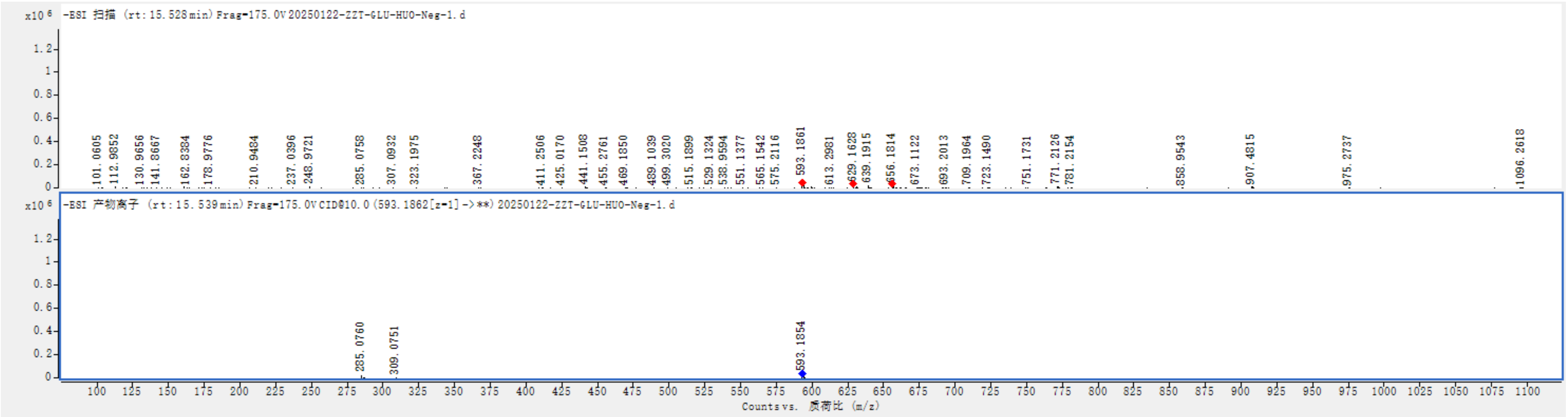

Supplementary Figure S3. Representative MS/MS spectrum of poncirin (negative ion mode).
